# Factors contributing to the mental wellbeing of Afghan migrants in Iran during the COVID-19 pandemic

**DOI:** 10.1016/j.jmh.2024.100211

**Published:** 2024-01-10

**Authors:** Fatemeh Khozaei, Qamar Ul Islam, T Ramayah, Nadia Ayub, Claus-Christian Carbon

**Affiliations:** a Department of Architectural Engineering, College of Engineering, Dhofar University, Salalah, Oman; b Department of Electrical Engineering, College of Engineering, Dhofar University, Salalah, Oman; c School of Management, Universiti Sains Malaysia, Minden, 11800 Penang, Malaysia; d Institute of Business Management Karachi, Pakistan; e Research Group EPÆG (Ergonomics, Psychological Aesthetics, Gestalt), Bamberg, Bavaria, Germany; f Department of General Psychology and Methodology, University of Bamberg, Bavaria, Germany; g Department of Information Technology & Management, Daffodil International University, Birulia, Bangladesh; h Department of Management, Sunway Business School (SBS), Petaling Jaya, 47500, Selangor, Malaysia; i University Center for Research & Development (UCRD), Chandigarh University, Ludhiana, 140413, Punjab, India; j Faculty of Economics and Business, Universitas Indonesia (UI), Depok City, 16424, West Java, Indonesia; k The University of Jordan (UJ), Aljubeiha, Amman, Jordan

**Keywords:** Mental health, Mental wellbeing, Migration, Stress, Depression, Social cohesion

## Abstract

This study aims to explore the factors contributing mental health of Afghan migrants residing in Iran during the COVID-19 pandemic. With a deep understanding of the unique challenges encountered by migrants, especially during times of crisis, this research delves into the influential factors of experienced anxiety, social cohesion, and stress and their significant contribution to the development of depression among Afghan migrants. The study included a sample of 469 individuals from the Afghan migrant community, aged 15 to 80 years. Data collection took place from December to March 2022 in Iran. The study revealed that anxiety and the burden of the COVID-19 pandemic significantly influenced the occurrence of depression among Afghan migrants. Furthermore, the relationship between these factors and depression was mediated by the experience of stress. Conversely, higher levels of perceived social cohesion in the host country were linked to reduced stress and depression among the migrants. As Afghan migrants in Iran face a heightened risk of developing depression, the importance of social support and integration is underscored by the association between higher levels of perceived social cohesion in the host country and reduced levels of stress and depression. Recognizing the vulnerabilities of this population, it becomes evident that fostering social support networks and promoting integration can play a crucial role in mitigating the negative impact of migration-related stressors and enhancing mental wellbeing among this population.

## Introduction

In recent years, there has been a significant increase in global migration, leading to a growing number of migrants who find themselves residing in foreign countries far away from their homeland. These individuals often encounter numerous challenges and obstacles that discourage them from considering a return to their home country. Migration is frequently motivated by a multitude of factors, including the persistent inequality between labour market supply and demand (Chernyak and Chernyak, 2019), as well as the presence of armed conflicts and safety concerns, encompassing persecution and violence (Mandić, 2021). Additionally, unfavourable economic conditions prevailing in the migrants’ countries of origin play a significant role in driving individuals to seek opportunities elsewhere (Ghatak, 2021).

Migration poses risks to both physical and mental health, which persist even after resettlement. Many migrants and refugees have endured distressing experiences in their home countries and throughout their migration trajectories (Giacco et al., 2018; Steel et al., 2017). Common mental disorders, including anxiety, post-traumatic stress, and depression, are prevalent among migrants (Awuah et al., 2022; Vukčević Marković et al. 2023; Nkimbeng et al., 2023; Lee et al., 2020). It seems that depression acts as a mediator in the relationship between migration stress, and sexual risk behaviours (Hou et al., 2019). Factors such as living in the host country for less than two years, insufficient family income, poor social support, and marital relationships were significantly associated with women migrants’ postpartum depression (PPD) (Xiong and Deng, 2020). Being female, unmarried, and having low satisfaction with the decision to migrate might increase the risk of screening positive for depression (See e.g., Aluh et al., 2023).

The emergence of the COVID-19 pandemic posed additional challenges for residents’ migrants and refugees worldwide (Khozaei et al., 2022a; Khozaei et al., 2022b; Khozaei and Carbon, 2022).The burden of COVID-19 was found to significantly affect immigrants’ stress and depression (Khozaei et al., 2022a). Several studies revealed that migrants, particularly those engaged in low-paid occupations, experienced psychological stressors associated with poverty (Espinel et al., 2020), discrimination and inequity (Li and Rose, 2020), social exclusion (Li and Jiang, 2020), and various unfavourable daily experiences during the COVID-19 crisis (Hou et al., 2020). Studies suggest that perceived justice and freedom in the host country can reduce migrants’ stress and depression, especially during the COVID-19 pandemic (Khozaei et al., 2022a). Previous studies suggest that social cohesion is associated with mental health in the migrant community (Cheung 2014; Qu et al., 2023) and can reduce migrants’ depression (Qu et al., 2023). There is a need to understand how social cohesion may affect the migrants' mental health during the COVID-19 era. Given the substantial population of Afghan migrants in Iran, this study focuses on Iran as a case study.

Afghan migrants, a significant population of migrants and refugees, have long confronted various challenges and disadvantages. The displacement of Afghan migrants has seen a significant surge in the past 40 years and notably, countries such as Iran, have emerged as key destinations for Afghan migrants and refugees in recent years. These individuals have endured traumatic experiences, long-term hardships, and the loss of loved ones, prompting their desperate escape from Afghanistan. The study examines the relationship between stress, anxiety, depression, and the burden of COVID-19 on these migrants. It further examines whether social cohesion affects these migrants’ mental health.

There are several rationales behind this research. First of all, the Afghan immigrant population residing in Iran largely consists of individuals engaged in temporary, low-paid jobs, making it difficult for many of them to afford mental health support. Hence, researching this minority group holds significant value in understanding the unique challenges Afghan migrants face in Iran. Second, it is essential to consider the possibility of future pandemics and their potential impact on migrant populations. Since the emergence of the COVID-19 pandemic, numerous studies have underscored the need to prepare for future pandemics. Understanding the mental health implications experienced by Afghan migrants during the current crisis can serve as a valuable resource for predicting and addressing mental health disorders that may arise in similar situations. By learning from past experiences, researchers can anticipate the potential mental health challenges that Afghan migrants and other migrant populations might encounter during future pandemics. Taking these proactive measures can enhance the resilience and wellbeing of Afghan migrants and similarly vulnerable populations when confronted with future-scale crises.

## Theoretical framework

Existing research indicates that Afghan migrants in different countries encounter significant stressors, including poverty, housing instability, and employment challenges (Kavian et al., 2020) that can affect their mental health. Factors contributing mental health of migrants are complex, especially during critical situations such as the COVID-19 pandemic (Alemi et al., 2023; Kurt et al., 2023). There are two ways to look at mental health, positive and negative. Despite their interdependence, these variables can function independently (Keyes, 2007). Positive mental health (PMH) is defined as optimal mental functioning and a sense of wellbeing. It has been conceptualized with various dimensions, such as eudaemonic and hedonic wellbeing (Keyes 2007). Negative mental health, on the other hand, refers to detrimental health issues, psychopathology, or psychiatric diseases.

In the last few years, several studies have been conducted on PMH (Vaganian et al., 2022; Velten et al., 2022) and how promoting PMH can affect individuals' wellbeing. Studies demonstrate that promoting positive mental health can mitigate the negative impact of insomnia symptoms on addictive online behavior (Brailovskaia et al., 2023). It is also connected to happiness and life satisfaction (Bieda et al., 2019) and may predict mental illness recovery (Iasiello et al., 2019). On the other hand, various factors might affect people's mental health negatively. Studies suggest that factors such as poverty (Chiavegatto Filho et al., 2013) particularly in areas characterized by income inequality, stress, and depression (Schönfeld et al., 2016) can affect an individual's mental health negatively.

Given its significance, numerous research studies have investigated elements that contribute to the psychological wellbeing of migrants amidst unique crises, such as the COVID-19 pandemic. This included but was not limited to economic limitations and job loss (Choudhari, 2020), heightened augmented daily stressors (Spiritus-Beerden et al., 2021), increased stress and anxiety levels (Palit et al., 2022; Jumaa et al., 2023) as well as perceived discrimination (Kurt et al., 2021). Several research studies have investigated the influence of factors that serve as buffers for the mental wellbeing of migrants amidst the COVID-19 pandemic which illuminate diverse protective mechanisms that play a role in alleviating the psychological hardships encountered by these individuals. For example, studies reveal that feeling of belonging and social connectedness can reduce the impact of perceived discrimination on mental health (Brance et al., 2022).

Afghan migrants residing in Iran, similar to migrants across different regions, confronted a distinct array of difficulties amid the COVID-19 pandemic. Considering the economic challenges, and lack of permanent employment of the majority of these migrants in Iran stemming from pre-existing vulnerabilities coupled with the added stressors imposed by the pandemic the study hypothesized that the Burden of COVID-19 and anxiety might affect the migrants' stress and depression. Drawing from earlier research indicating stress as a potential precursor to depression (Sun et al., 2020), it was also postulated that heightened stress among migrants could elevate their susceptibility to experiencing depression amid the COVID-19 pandemic.

Numerous studies have highlighted the essential connection between justice and social cohesion. The works of Schiefer and van der Noll (2017), Laurence (2011), and Correa-Velez et al. (2017) revealed that perceived discrimination and inequality profoundly affected social solidarity among minority groups in the Netherlands, the UK, and refugee populations in Australia. These studies found that experiences of injustice and exclusion not only weakened community bonds but also hindered the smooth adaptation of these groups into society. They emphasized the crucial role of fairness and equity in facilitating the integration of marginalized communities. Overall, these findings underline the critical importance of justice in fostering social cohesion, particularly for marginalized groups, highlighting the necessity of inclusive policies and practices that ensure the respect and integration of minority populations. Fonseca et al. (2019) further emphasized the pivotal role of justice as the foundation for upholding social cohesion, stressing the significance of equitable treatment in creating an environment that is inclusive and supportive for Afghan migrants.

Research underscores a strong correlation between social cohesion, freedom and sense of belonging. It was found that restricted individual autonomy and liberties lead to the isolation of vulnerable groups from the larger society (Bilgili et al., 2017; Alanya et al., 2017). In contrast, the presence of unrestricted participation fosters stronger communal bonds and identity in various contexts (Staver, 2013; Rapp and Freitag, 2015). These findings emphasize the crucial role of liberty in nurturing unified and inclusive societies that embrace diversity. Consequently, our understanding of social cohesion incorporates the notions of belonging and freedom, rooted in empirical evidence. Empowerment and independence are pivotal for building meaningful connections, particularly for marginalized populations.

**Fig. 1:**
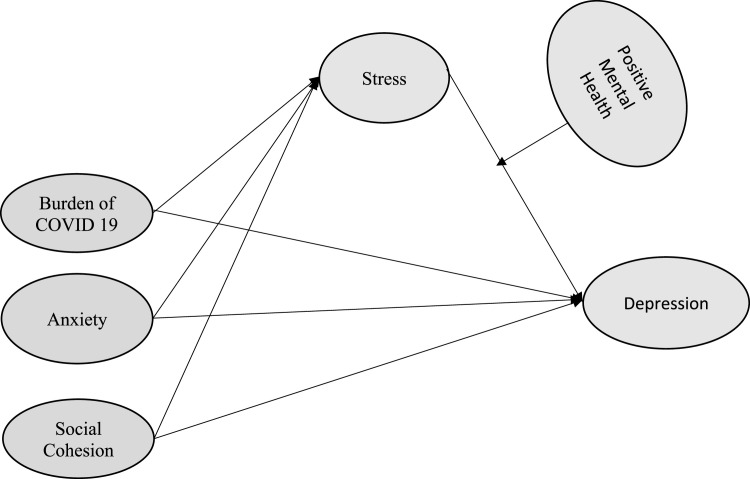
Research conceptual model.

Research conducted in the past indicates that social cohesion has the potential to benefit individuals’ mental wellbeing (An et al., 2023). Social cohesion epitomizes the interconnected unity within societal groups and holds a significant connection to the ideals of justice freedom and a sense of belonging (Manca, 2014). In addition, it is suggested that individuals who believe they have been treated fairly and have a sense of freedom within their communities may experience better mental health outcomes (Scholten et al., 2017). A growing body of literature has demonstrated a positive relationship between mental health and a belief in the existence of justice within societies. Perceptions of justice are often associated with social and macroeconomic factors (Fischer, 2016) and have been linked to various health benefits (Sert et al., 2014). Taking into account the perceived feelings of belonging, justice, and freedom within the context of social cohesion in the host nation as compared to their home country, the study further conjectured that enhanced social cohesion could enhance the mental wellbeing of these individuals by reducing stress and depression. Fig. 1 presents the conceptual model of research. Based on the aforementioned concepts, the research hypotheses are presented below:

• H1: The burden of COVID-19 is indirectly linked to depression through the mediating role of stress.
• H2: Anxiety indirectly affects depression through the mediating role of stress.
• H3: Social cohesion indirectly influences depression through the mediating role of stress.
• H4: The relationship between stress and depression is moderated by positive mental health.

## Research methodology

In this study, we aimed to investigate the factors influencing mental health among Afghan migrants in Iran. Specifically, we sought to explore the relationship between stress, depression, the burden of COVID-19, and perceived social cohesion. There is a lack of comprehensive data regarding the mental health status of Afghan migrants residing in the southern region of Iran.

### Participants

According to the statistical centre of Iran's report in 2015, the majority of Afghan migrants reside in the provinces of Kerman, Tehran, Khorasan, and Isfahan. For our study, participants were recruited from the city of Kerman, Iran, among Afghan migrants. The target population consisted of individuals aged 15 and above who originated from Afghanistan or were born in Iran but did not possess citizenship or equal rights as local Iranians. Kerman is home to over 40,000 Afghan migrants. Using the Cochran formula to determine the appropriate sample size based on desired precision and confidence level, a minimum of 380 participants was required. To bolster the robustness of the findings, a decision was made to administer more questionnaires, surpassing the initially calculated sample size. This choice was driven by the need to account for potential non-response and incomplete surveys, which are commonly encountered in survey-based research. Through the utilization of a larger sample size, the aim was to attenuate the potential bias introduced by missing data and non-response, consequently elevating the reliability and validity of our results.

### Measurement scale

The questionnaire distributed in this study encompassed various items and constructs to assess different aspects. The initial section of the questionnaire aimed to gather demographic information from the respondents, including their gender, age, marital status and duration of stay. To measure stress and depression, we utilized the Depression, Anxiety, and Stress Scale (DASS-21), a well-established scale frequently employed in assessing mental health. The DASS-21 has consistently demonstrated robust construct and structural validity across diverse nations (see, e.g., Bibi et al., 2020). Participants were asked to respond to 21 questions relating to their stress and depression and anxiety experiences over the past seven days. The responses could be given on scale from 1 (*didn't apply to me at all*) to 4 (*applied to me very much or most of the time*)*.*

The burden of COVID-19 was measured using 10 items adapted from the research conducted by Brailovskaia and Margraf (2021). The responses aimed to assess individuals' understanding and encounter of the societal and personal ramifications of the pandemic on their lives. The items encompassed a spectrum of emotions, viewpoints, and perspectives linked to the pandemic circumstances. The responses could be given f*rom, I do not agree* to *7 I totally agree.*

For measuring positive mental health PMH scale was used (Lukat et al., 2016). 9 items measured collectively encompassed aspects of an individual's positive psychological wellbeing and mental health. The questions seeked to gauge the individual's overall positive outlook, contentment, and ability to cope with various life situations. The responses could be given from 1 (*do not agree*) to 4 (*totally agree*). To measure social cohesion three items from (Scholten et al., 2017, 2018). The items aimed to gauge individuals’ sense of fitting into their social environment, their assessment of equitable treatment, and their perceived degree of liberty within their native country in comparison to their home country. The responses could be given from 0 (*not at all*) to 100 (*very much on each statement*). Table 1 provides an overview of the measurement constructs and specific items used in the questionnaire.

**Table 1:** Measurement constructs and items.

Construct	Items	Questions	Measurement
Burden of COVID-19	BUC1	The current social situation burdens me	1 = *I do not agree* 7 = *I totally agree*
	BUC2	I feel restricted in my everyday life	
	BUC3	I am afraid of the current situation	
	BUC4	I feel socially isolated	
	BUC5	I'm making the best of the situation	
	BUC6	I look forward to what will happen	
	BUC7	I now know better what is important to me	
	BUC8	My relationships with family and friends have become stronger and better	
	BUC9	My current situation has some advantages	
	BUC10	I am worried about my future life	
Depression	DEP1		1 = *did not apply to me at all* 4 = *applied to me very much, or most of the time*
	DEP1	I was unable to become enthusiastic about anything	
	DEP2	I was aware of the dryness of my mouth	
	DEP3	I couldn't seem to experience any positive feelings at all	
	DEP4	I felt that I was rather touchy	
	DEP5	I found it difficult to work up the initiative to do things	
	DEP6	I tended to over-react to situations	
	DEP7	I felt that life was meaningless	
Stress	STR 1	I felt I was close to panic	1 = *did not apply to me at all* 4 = *applied to me very much, or most of the time*
	STR2	I experienced breathing difficulty (e.g., excessively rapid breathing, breathlessness in the absence of physical exertion)	
	STR3	I felt I wasn't worth much as a person	
	STR4	I found it hard to wind down	
	STR5	I was aware of the action of my heart in the absence of physical exertion (e.g., sense of heart rate increase, heart missing a beat)	
	STR6	I felt scared without any good reason	
	STR7	I experienced trembling (e.g., in the hands)	
Social Cohesion		When you compare yourself with other people in 〈your home country〉, how belonging do you feel to this society?	*0 = not at all socially belonging* *100 = very socially belonging*
		When you compare yourself with other people in 〈your home country〉, how **fairly** treated do you feel?	*0 = not fair at all 100 = very fair*
		When you compare yourself with other people in 〈your home country〉, how **free** do you feel?	*0 = not free at all 100 = very free*
Anxiety		I was aware of the dryness of my mouth.	*0 = did not apply to me at all* 3 = applied to me very much, or most of the time
		I experienced trembling (e.g., in the hands).	
		I felt that I was using a lot of nervous energy.	
		I felt that I was worrying too much about different things.	
		I found myself getting agitated.	
		I found it difficult to relax.	
		I felt scared without any good reason.	
Positive Mental Health		I am often carefree and in good spirits.	*1 = do not agree, 4 = totally agree*
		I enjoy my life.	
		All in all, I am satisfied with my life.	
		In general, I am confident.	
		I manage well to fulfil my needs.	
		I am in good physical and emotional condition.	
		I feel that I am actually well equipped to deal with life and its difficulties.	
		Much of what I do brings me joy.	
		I am a calm, balanced human being.	

### Procedure

A multi-stage sampling strategy was used to recruit the sample. In stage 1, the researchers targeted Kerman, Iran, as the capital of one of the provinces with the largest number of Afghan migrants. In stage 2, three districts with a high proportion of Afghan migrants were chosen. In stage 3, the neighborhoods with the largest population of these migrants were identified. During the field visit and on-site data collection, aided by local Afghan residents in each neighborhood, the researchers engaged with Afghan migrant families, requesting their participation in our survey. To select participants, we employed a convenience sampling technique within the chosen neighborhoods, reaching out to individuals through connections with local residents. Notably, 65 families opted to decline involvement in the study. Please note that we deliberately utilized a convenience sampling technique for the following reasons: Convenience sampling is particularly useful when working with participants with a challenging background, such as refugees, for several reasons. Firstly, it involves selecting participants who are readily available and willing to participate, which can be less intrusive and stressful for individuals who may have experienced trauma or are in vulnerable situations. This approach also provides easier access to these populations, who may be difficult to reach through traditional sampling methods. Building trust is crucial with these groups, and convenience sampling often occurs in environments where participants feel safe and supported, like community centers or support groups. This facilitates better engagement and honest responses. Moreover, convenience sampling allows researchers to adhere to strict ethical guidelines, respecting the privacy and confidentiality of participants and reducing the risk of re-traumatization or exposure to harm. It also enables greater cultural sensitivity, as researchers can employ “interpreters” or “cultural mediators” familiar to the participants, ensuring better communication and understanding. While convenience sampling has practical and ethical advantages, we are also aware of potential limitations in the representativeness and generalizability of findings.

Face-to-face encounters were used for recruitment. Before the commencement of data collection, all participants were furnished with a consent form. This form explicitly outlined the research's objectives and emphasized the confidentiality measures in place to protect their information. During the continuing COVID-19 epidemic in Iran, data was collected over four months to record differences in experiences and perceptions of mental health. Researchers kept in touch with participants frequently, addressing any issues or queries that emerged during the data-gathering procedure.

Between December and March 2022, we collected the data for the present study in Iran. The data collection process was conducted by two research assistants, one Iranian and one Afghan-trained—this elaborate procedure was used to optimally assist the participants regarding language and empathy. Before participating, all respondents were provided with a consent form explaining the research objectives and assured that their responses would be kept confidential. The questionnaire included inquiries about participants’ demographic background, perceived stress, depression experienced within the past seven days, and the burden of COVID-19. Upon completion of the survey, participants received a token of appreciation as a gesture of gratitude.

To analyse the research model and test the hypotheses, Partial Least Squares (PLS) analysis was conducted using the SmartPLS 3.3 software (Ringle et al., 2015). Given the exploratory nature of the study and the presence of hierarchical variables, PLS was deemed appropriate for the analysis. To determine the significance of the path coefficients among the latent variables, a nonparametric bootstrapping technique with 5000 iterations was employed. Furthermore, the reliability and validity of the measurement model, as well as the structural model, were assessed to ensure the robustness of the findings. Construct reliabilities and validities were evaluated using measures such as rho_A, Composite Reliability (*CR*), *CA*, and Average Variance Extracted (*AVE*). The assessment of the structural model involved testing and reporting coefficients of determination (*R*^2^), effect size (*f*^2^), variance inflation factor (*VIF*), and predictive relevance (*Q2*).

### Ethics

Before the commencement of data collection, all participants were given a consent form. This form explicitly outlined the research's objectives and emphasized the confidentiality measures in place to protect their information. To check the ethics of the study, especially the involvement of minors (we tested participants aged 15 and above), we asked the first author's current institution for approval. We obtained clearance for ethical approval and data collection from the University Research Ethics & Biosafety Committee, the responsible local ethics committee of the first author's institution (protocol-nr #DU-AY-23-24-QUES-012).

## Results

Data was collected from five districts with the highest population of Afghan migrants, and participants were proficient in the Farsi language. Out of the 497 questionnaires administered and collected, 28 were excluded due to incomplete responses, resulting in a total of 469 questionnaires suitable for analysis. Table 2 presents the demographic information of the respondents who took part in the study. The average age of participants was 30.6 years (*SD*= 14.30). Of the participants, 191 were male and 278 were female. In terms of age groups, 362 participants were aged between 15 and 35 years, 62 were between 36 and 55 years old, and 45 were above 45 years old. Among the participants, 169 indicated to be single and 300 reported to be married.

**Table 2:** Respondents’ demographic characteristics.

Demographic factors	Categories	*N*	Percentage
Gender	Male	191	40.7 %
	Female	278	59.3 %
Age	15–35 years	362	77.1 %
	36–55 Years	62	13.2 %
	above 56 years	45	9.6 %
Marriage status	single	169	36.0 %
	married	300	64.0 %
Duration of living in Iran	up to 10 years	62	13.7 %
	11–20 years	190	19.8 %
	21–30 years	127	50.0 %
	31–40 years	60	13.2 %
	above 40 years	15	3.3 %

### Measurement model assessment

The proposed measurement model was assessed and employed a two-stage approach as suggested by Anderson and Gerbing (1988). First, we assessed the measurement model for each latent variable undertaken in the study. For the study, we conceptualized the measurement model as a reflective measurement model, which allows us to assess the reliability, the convergent and discriminant validity. The reliability of the measurement model was assessed by employing Cronbach's alpha coefficient, and the Composite Reliability (CR) with the cut-off value of 0.70 is acceptable. Initially, the overall model was assessed, and constructs with AVE lower than 0.50 were identified.

Items with the lowest loadings were discarded. Table 3 indicates that the value of CR and Cronbach's alpha exceeded the cut-off value of 0.70, suggesting internal consistency reliability. As evaluated by the value of average variance extracted (AVE) for all of the constructs, the convergent validity exceeded the cut-off value of 0.50. Besides the outer loading value of all items of each construct: Burden of COVID-19 (BUC), Anxiety (Anxie) Depression (Depr), Social Cohesion (Soci), positive mental health (PMH) and stress (STR) is higher than 0.70. Table 3 presents the established reliability and convergent validity of research constructs.

**Table 3:** Reliability and convergent validity for research constructs.

Construct	Item	Loading	rho_A	*CR*	*CA*	*AVE*
Burden of COVID-19	BUC1	0.869	0.862	0.895	0.845	0.682
	BUC2	0.768				
	BUC4	0.811				
	BUC9	0.851				
Anxiety	Anxie1	0.515	0.908	0.913	0.887	0.606
	Anxie2	0.812				
	Anxie3	0.856				
	Anxie4	0.798				
	Anxie5	0.829				
	Anxie6	0.697				
	Anxie7	0.881				
Depression	Depr1	0.880	0.946	0.956	0.946	0.755
	Depr2	0.864				
	Depr3	0.852				
	Depr4	0.862				
	Depr5	0.901				
	Depr6	0.863				
	Depr7	0.858				
Social Cohesion	Soci1	0.474	0.726	0.762	0.596	0.530
	Soci2	0.776				
	Soci3	0.874				
Stress	Stress1	0.917	0.943	0.952	0.941	0.741
	Stress2	0.829				
	Stress3	0.824				
	Stress4	0.835				
	Stress5	0.832				
	Stress6	0.873				
	Stress7	0.911				
Positive Mental Health	PMH1	0.915	0.876	0.862	0.796	0.830
	PMH2	0.846				
	PMH3	0.912				
	PMH4	0.853				
	PMH5	0.916				
	PMH6	0.906				
	PMH7	0.874				
	PMH8	0.740				

CR = composite reliability; AVE = average variance extracted; VIF = variance inflation factor.

### Structural model assessment and multigroup analysis

#### Direct effects

The result of the study showed a significant positive effect of the Burden of COVID-19 on Stress (β = 0.036, *p* = 0.001), suggesting that migrants who experienced a higher burden of COVID-19 tend to have increased levels of stress. Besides anxiety had a strong positive effect on stress levels (β = 0.900, *p* = 0.042). The results highlight the effect of social Cohesion on Stress (β = −0.071, *p* = 0.037). The negative path coefficient suggests that higher levels of social cohesion are associated with lower levels of stress. The result also highlighted the effect of Stress on Depression (β = 0.521, *p* < 0.001). This indicates that higher stress levels are positively associated with increased levels of depression. The effect of the Burden of COVID-19 on Depression (β = 0.089, *p* < 0.001). This suggests that individuals facing a greater burden of COVID-19 are more likely to experience higher levels of depression. The results highlight the effect of Anxiety on Depression (β = 0.317, *p* = 0.018). This indicates that individuals with higher levels of anxiety are more likely to experience increased levels of depression. The results indicate a marginally significant effect of Social Cohesion on Depression (β = −0.088, *p* = 0.067). The negative path coefficient suggests that higher levels of social cohesion are associated with lower levels of depression. However, the p-value of 0.067 is marginally significant (*p* < 0.1), indicating that more data or further investigation may be needed to establish the strength of this relationship more conclusively.

Based on the *R^2^* values, anxiety, burden of COVID-19 and social cohesion approximately explained 91 % of the variance of depression and 84 % of the variance of the stress. The effect size (*f^2^*) estimates how much an IV affects DV. As suggested by Chin (1998), the values of 0.02, 0.15, and 0.35 represent the level of effect size as small, moderate, and substantial, respectively. The effect of the burden of COVID-19 on stress (*f^2^* = 0.142), and stress on depression (*f^2^*= 0.420) is considered moderate, indicating a moderate impact of the COVID-19 burden on stress levels. The effect of anxiety on stress (*f^2^*= 1.353) is considered substantial, suggesting a significant influence of anxiety on stress levels. The effect of social cohesion on stress (*f^2^*= 0.045), the burden of COVID-19 on depression (*f^2^*= 0.042), and anxiety on depression (*f^2^*= 0.180), The effect of social cohesion on depression (*f^2^*= 0.041) is considered small, indicating a relatively minor impact of social cohesion on stress.

Multicollinearity among the variables was evaluated, considering the variance inflation factor (*VIF*) value. It is suggested that *VIF* that exceeds 10 indicates a potential issue with multicollinearity (Hair et al., 2010). As shown in Table 4, VIF for all of the variables was below the threshold of 10. The predictive relevance of the model (*Q^2^*) was examined with the help of the blindfolding procedure. The *Q^2^* values for depression (0.823) and stress (0.765) suggested that the model has sufficient predictive relevance.

**Table 4:** Direct effects.

Direct/indirect effect	Path Coefficient *β*	*t-*value	*p-*value	*VIF*	*f^2^*	Supported
The Burden of COVID-19 Stress	0.036	3.234	<0.001	1.999	0.142	Yes
Anxiety → Stress	0.900	2.834	0.042	2.925	1.353	Yes
Social Cohesion → Stress	−0.071	1.754	0.037	2.223	0.045	Yes
Stress → Depression	0.521	4.417	<0.001	4.925	0.420	Yes
Burden of COVID-19 → Depression	0.089	3.722	<0.001	2.282	0.042	Yes
Anxiety → Depression	0.317	2.113	0.018	4.881	0.180	Yes
Social Cohesion → Depression	−0.088	1.634	0.067	2.324	0.041	Yes

#### Indirect effect

**Fig. 2:**
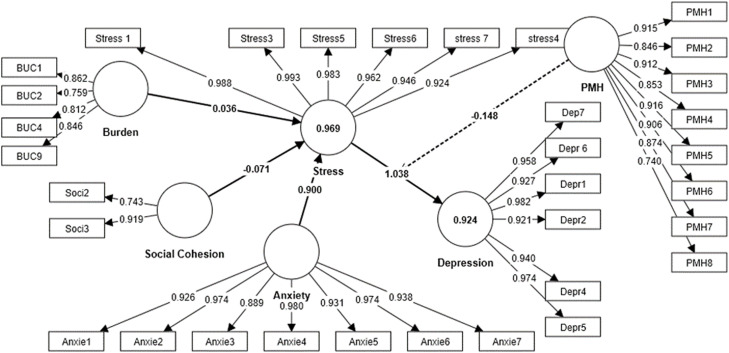
structural equation model (SEM) diagram.

In this study, we examined the mediating effect of stress on the relationship between the burden of COVID-19, anxiety social cohesion, and depression. For examining the possible mediating effect of stress, we applied the *nts* approach (indirect effect). The indirect effect was assessed with the help of a bootstrapping procedure for examining the possible significance of the path coefficients (Chin et al., 2008). Table 5 shows the results of the path analysis used to test the hypothesis of indirect effects. The *t*-values were computed using a bootstrapping procedure (Hayes, 2009) with 5000 samples. Hypothesis 1 investigated the mediating role of stress in the relationship between the burden of COVID-19 and depression. The findings confirmed this mediating effect (β =0.098, *t* = 6.105, *p* < 0.001). Hypothesis 2 examined the mediating effect of stress between anxiety and depression (β =0.367, *t* = 9.624, *p* < 0.001). Hypothesis 3 was also supported as stress mediated the relationship between social cohesion and depression (β = −0.059, *t* = 3.586, *p* < 0.01). Hypothesis 4 hypothesized the moderating effect of positive mental health on the effect of stress on depression. This hypothesis was also supported (β = −0.148, *t* = 1.586, *p <* 0.001). Fig. 2 presents the research model.

**Table 5:** Structural (inner) model assessment.

Hypothesis	Indirect Effect	Path Coefficient	Confidence interval (95 %) Bias Corrected	*t-*value	*p*-value	Supported
H1	BUC -> Stress -> Depression	0.098	0.124	6.105	0.002	Yes
H2	Anxiety> Stress -> Depression	0.367	0.432	9.624	<0.001	Yes
H3	Social Cohestion> Stress > Depression	−0.059	0.034	3.586	<0.001	Yes
H4	Stress->Positive mental health-> depression	−0.148	0.234	1.586	<0.001	Yes

## Discussion

This study aims to explore the intricate relationship between migration and depression experienced by Afghan migrants residing in Iran during the COVID-19 pandemic. With a deep understanding of the unique challenges encountered by migrants, especially during times of crisis, this research delves into the influential factors of experienced anxiety, social cohesion, and stress, and their significant contribution to the development of depression among Afghan migrants.

First, the findings revealed a significant positive effect of the burden of COVID-19 on stress, indicating that migrants who experienced a higher burden of COVID-19 tended to have increased levels of stress. This result aligns with previous research highlighting the psychological impact of the pandemic on individuals’ wellbeing (Luo et al., 2020). It suggests that the challenges and uncertainties associated with COVID-19 contribute to elevated stress levels among immigrants.

Furthermore, anxiety exhibited a strong positive effect on stress levels, emphasizing the influence of anxiety on individuals' psychological wellbeing. This finding supports the existing literature (Amini et al., 2022) linking anxiety to higher levels of stress and reflects the psychological strain experienced by individuals with elevated anxiety levels.

Social cohesion (Silveira et al., 2022) emerged as a significant factor affecting stress levels. The negative path coefficient suggests that higher levels of social cohesion are associated with lower levels of stress. This finding underscores the importance of social support and community connections in mitigating stress among immigrants. It implies that fostering social cohesion and community engagement may serve as protective factors for individuals facing stressors, such as the burden of COVID-19 and anxiety. It further suggests that an increased level of freedom promotes better mental health.

Consistent with previous research (Werner et al., 2020), the results indicate that stress is positively associated with depression. Individuals experiencing higher levels of stress are more likely to report increased levels of depression. This finding highlights the detrimental impact of stress on mental health outcomes and emphasizes the need to address stress management and coping strategies to mitigate depression symptoms.

The burden of COVID-19 also exhibited a significant positive effect on depression, suggesting that individuals facing a greater burden of COVID-19 (McQuaid et al., 2021) are more likely to experience higher levels of depression. This finding aligns with the notion that the pandemic's consequences, such as health risks, economic instability, and social disruptions, can contribute to adverse mental health outcomes, including depression.

Similarly, anxiety was found to have a significant positive effect on depression, indicating that individuals with higher anxiety levels are more susceptible to experiencing increased levels of depression (Jia et al., 2023). This result supports the well-established link between anxiety and depression and underscores the need to address both anxiety and depression symptoms in interventions and support systems.

The effect of social cohesion on depression showed a negative path coefficient, suggesting that higher levels of social cohesion are associated with lower levels of depression. Although the p-value of 0.067 is marginally significant (*p* < 0.1), indicating the need for further investigation (Biddle et al., 2020), this finding suggests that social cohesion may serve as a protective factor against depression. Strengthening social support networks, fostering community connections, and promoting a sense of belonging may contribute to improved mental wellbeing and lower levels of depression.

The R2 values indicated that anxiety, the burden of COVID-19, and social cohesion collectively explained a substantial amount of the variance in depression and stress (Marshall et al., 2022). This suggests that these factors play significant roles in influencing mental health outcomes in the studied population.

Furthermore, the effect size (*f*^2^) values provided insights into the magnitude of the relationships. The *f*^2^ values indicated that the burden of COVID-19 on stress, stress on depression, and anxiety on stress had moderate to substantial effect sizes. This implies that these factors have noteworthy impacts on mental health outcomes. On the other hand, the *f*^2^ values for social cohesion on stress, burden of COVID-19 on depression, anxiety on depression, and social cohesion on depression were smaller, suggesting relatively minor impacts of these factors.

## Conclusion

COVID-19 brought about a momentous event in the past few decades, leaving an indelible mark on our era. The alterations it introduced to the everyday rhythms of global life caught many off guard, resulting in a challenging process of adjustment. Both migrants and local residents experienced unique mental health challenges during this time. However, the findings from our study emphasize the pivotal role the host country can play in enhancing their psychological wellbeing. Specifically, focusing on social cohesion emerges as a potent measure to alleviate the stress and depression that migrants often grapple with, ultimately contributing to an overall enhancement in their mental health.

The study's findings have crucial implications for policy and practice in improving Afghan migrants’ mental health and general wellbeing. Policymakers should prioritize the development of tailored treatments to alleviate the COVID-19 burden, anxiety, and stress in this group. Culturally appropriate mental health treatments, greater access to healthcare, and financial stability should all be priorities. Furthermore, community-based initiatives promoting social cohesiveness, safe spaces for interaction, and a sense of belonging might act as protective factors against depression and lead to better mental health outcomes.

While this study provides important insights into the complex interaction between migration, mental health, and the burden of COVID-19 among Afghan migrants, further research is needed in this area. Future research should delve deeper into the experiences and mental health needs of Afghan migrants, taking into account aspects such as economic challenges. Longitudinal studies can give a thorough knowledge of migration and the pandemic's long-term consequences on mental health outcomes. Such a study can help to influence the development of evidence-based therapies and policies that address the mental health difficulties that Afghan migrants experience.

Besides our use of a convenience sampling, we focused on a specific neighborhoods in Kerman, which may not fully represent the diverse Afghan migrant population, given the lack of randomization and access to isolated members. Hence, the generalizability of our findings to the broader Afghan migrant community in Kerman and Iran is of course limited. Future studies should employ systematic, randomized sampling methods to ensure a more comprehensive understanding of this population, but in a given situation of a crisis, such methodological sophistication is often not fully implementable.

## CRediT authorship contribution statement

**Fatemeh Khozaei:** Conceptualization, Data curation, Formal analysis, Methodology, Project administration, Resources, Software, Validation, Visualization, Writing – original draft, Writing – review & editing. **Qamar Ul Islam:** Writing – review & editing. **T Ramayah:** Writing – review & editing. **Nadia Ayub:** Writing – review & editing. **Claus-Christian Carbon:** Supervision, Writing – review & editing, Conceptualization, Funding acquisition, Visualization, Writing – original draft.

## Declaration of competing interest

No conflict of interest exists.

We wish to confirm that there are no known conflicts of interest associated with this publication and there has been no significant financial support for this work that could have influenced its outcome.

## References

[bib0001] Alanya A., Swyngedouw M., Vandezande V., Phalet K. (2017). Close encounters: minority and majority perceptions of discrimination and intergroup relations in Antwerp, Belgium. Int. Migrat. Rev..

[bib0002] Alemi Q., Panter-Brick C., Oriya S., Ahmady M., Alimi A.Q., Faiz H., Ventevogel P. (2023). Afghan mental health and psychosocial wellbeing: thematic review of four decades of research and interventions. BJPsych. Open..

[bib0005] Aluh D.O., Aigbogun O., Anyachebelu O.C. (2023). Depression among immigrant Nigerians in Canada. J. Immigr. Minor. Health.

[bib0003] Amini B., Raheel O., Exum A., Fazzino T.L. (2022). Mental health of Iranian migrants and their descendants: a review. Profess. Psychol.: Res. Pract..

[bib0004] An S., Kim K., Lee M. (2023). Longitudinal relationships between social cohesion, mental health and functional disabilities in later life. Educ. Gerontol..

[bib0006] Anderson J.C., Gerbing D.W. (1988). Structural equation modeling in practice: a review and recommended two-step approach. Psychol. Bull..

[bib0007] Awuah R.B., de-Graft Aikins A., Dodoo F.N.-A., Meeks K.A., Beune E.J., Klipstein-Grobusch K., Addo J., Smeeth L., Bahendeka S.K., Agyemang C (2022). Psychosocial stressors among Ghanaians in rural and urban Ghana and Ghanaian migrants in Europe. J. Health Psychol..

[bib0008] Biddle N., Edwards B., Gray M., Sollis K. (2020).

[bib0009] Bibi A., Lin M., Zhang X.C., Margraf J. (2020). Psychometric properties and measurement invariance of Depression, Anxiety and Stress Scales (DASS-21) across cultures. Int. J. Psychol..

[bib0010] Bieda A., Hirschfeld G., Schönfeld P., Brailovskaia J., Lin M., Margraf J. (2019). Happiness, life satisfaction and positive mental health: investigating reciprocal effects over four years in a Chinese student sample. J. Res. Pers..

[bib0011] Bilgili Ö., Huddleston T., Joki A.L. (2017).

[bib0012] Brailovskaia J., Margraf J. (2021). The relationship between burden caused by a coronavirus (Covid-19), addictive social media use, sense of control, and anxiety. Comput. Human Behav..

[bib0013] Brailovskaia J., Balcerowska J.M., Precht L.M., Margraf J. (2023). Positive mental health mediates the association between insomnia symptoms and addictive social media use in Germany and Poland. Comput. Human Behav..

[bib0014] Brance K., Chatzimpyros V., Bentall R.P. (2022). Perceived discrimination and mental health: the role of immigrant social connectedness during the COVID-19 pandemic. J. Migr. Health.

[bib0015] Cheung N.W. (2014). Social stress, locality of social ties and mental wellbeing: the case of rural migrant adolescents in urban China. Health Place.

[bib0016] Choudhari R. (2020). COVID 19 pandemic: mental health challenges of internal migrant workers of India. Asian J. Psychiatr..

[bib0017] Chernyak O., Chernyak Y. (2019). SHS Web of Conferences.

[bib0018] Chiavegatto Filho A.D.P., Kawachi I., Wang Y.P., Viana M.C., Andrade L.H.S.G (2013). Does income inequality get under the skin? A multilevel analysis of depression, anxiety, and mental disorders in São Paulo, Brazil. J. Epidemiol. Community Health.

[bib0019] Chin W.W. (1998). Modern Methods for Business Research.

[bib0020] Chin W.W., Peterson R.A., Brown S.P. (2008). Structural equation modeling in marketing: some practical reminders. J. Market. Theory Pract..

[bib0021] Correa-Velez I., Gifford S.M., McMichael C., Sampson R. (2017). Predictors of secondary school completion among refugee youth 8 to 9 years after resettlement in Melbourne, *Australia*. Int. Migrat..

[bib0022] Espinel Z., Chaskel R., Berg R.C., Florez H.J., Gaviria S.L., Bernal O. (2020). Venezuelan migrants in Colombia: COVID-19 and mental health. Lancet Psychiatry.

[bib0023] Fischer R., Sabbagh C., Schmitt M. (2016). Handbook of Social Justice Theory and Research.

[bib0024] Fonseca X., Lukosch S., Brazier F. (2019). Social cohesion revisited: a new definition and how to characterize it. Innovation: Eur. J. Soc. Sci. Res..

[bib0025] Ghatak S. (2021). Urban Growth and Environmental Issues in India.

[bib0026] Giacco D., Laxhman N., Priebe S. (2018). Prevalence of and risk factors for mental disorders in refugees. Semin. Cell Dev. Biol..

[bib0027] Hair J.F., Black B., Babin B.J., Anderson R.E. (2010).

[bib0028] Hayes A.F. (2009). Beyond Baron and Kenny: statistical mediation analysis in the new millennium. Commun. Monogr..

[bib0029] Hou B., Nazroo J., Banks J., Marshall A. (2019). Impacts of migration on health and well-being in later life in China: Evidence from the China Health and Retirement Longitudinal Study (CHARLS). Health  Place.

[bib0030] Hou W.K., Liu H., Liang L., Ho J., Kim H., Seong E. (2020). Everyday life experiences and mental health among conflict-affected forced migrants: a meta-analysis. J. Affect. Disord..

[bib0039] Iasiello M., van Agteren J., Keyes C.L., Cochrane E.M. (2019). Positive mental health as a predictor of recovery from mental illness. J. Affect. Disord..

[bib73] Jia S., Hou Y., Wang D., Zhao X. (2023). Flavonoids for depression and anxiety: a systematic review and meta-analysis. Crit. Rev. Food Sci. Nutr..

[bib0031] Jumaa J.A., Bendau A., Ströhle A., Heinz A., Betzler F., Petzold M.B. (2023). Psychological distress and anxiety in Arab refugees and migrants during the COVID-19 pandemic in Germany. Transcult. Psychiatry.

[bib0032] Kavian F., Mehta K., Willis E., Mwanri L., Ward P., Booth S. (2020). Migration, stress and the challenges of accessing food: an exploratory study of the experience of recent Afghan women refugees in Adelaide, Australia. Int. J. Environ. Res. Public Health.

[bib0033] Kurt G., Ekhtiari M., Ventevogel P., Ersahin M., Ilkkursun Z., Akbiyik N., Acarturk C. (2023). Socio-cultural integration of Afghan refugees in Türkiye: the role of traumatic events, post-displacement stressors and mental health. Epidemiol. Psychiatr. Sci..

[bib0034] Keyes C.L.M. (2007). Promoting and protecting mental health as flourishing: a complementary strategy for improving national mental health. Am. Psychol..

[bib0035] Khozaei F., Carbon C.-C., Abd Razak N (2022). Determinants of mental disorders of Afghan migrants during the COVID-19 pandemic. Int. J. Migrat., Health Soc. Care.

[bib0036] Khozaei F., Carbon C.C., Hosseini Nia M., Kim M.J. (2022). Preferences for hotels with biophilic design attributes in the post-COVID-19 era. Buildings.

[bib0037] Khozaei F., Carbon C.C. (2022). On the parental influence on children's physical activities and mental health during the COVID-19 pandemic. Front. Psychol..

[bib0038] Kurt G., Ilkkursun Z., Javanbakht A., Uygun E., Karaoglan-Kahilogullari A., Acarturk C. (2021). The psychological impacts of COVID-19 related stressors on Syrian refugees in Turkey: the role of resource loss, discrimination, and social support. Int. J. Intercult. Relat..

[bib0040] Laurence J. (2011). The effect of ethnic diversity and community disadvantage on social cohesion: a multi-level analysis of social capital and interethnic relations in UK communities. Eur. Sociol. Rev..

[bib0041] Lee M., Bhimla A., Ma G.X. (2020). Depressive symptom severity and immigration-related characteristics in Asian American Immigrants. J. Immigr. Minor Health.

[bib0042] Lukat J., Margraf J., Lutz R., van der Veld W.M., Becker E.S. (2016). Psychometric properties of the positive mental health scale (PMH-scale). BMC Psychol..

[bib0043] Luo M., Guo L., Yu M., Jiang W., Wang H. (2020). The psychological and mental impact of coronavirus disease 2019 (COVID-19) on medical staff and general public - a systematic review and meta-analysis. Psychiatry Res..

[bib0044] Li C., Jiang S. (2020). Social exclusion, sense of school belonging and mental health of migrant children in China: a structural equation modeling analysis. Child Youth Serv. Rev..

[bib0045] Li J., Rose N. (2020). Urban social exclusion and mental health of China's rural-urban migrants: a review and call for research. Health Place.

[bib0046] Manca A.R., Michalos A.C. (2014). Encyclopedia of Quality of Life and Wellbeing Research.

[bib0047] Mandić D. (2021). What is the force of forced migration? Diagnosis and critique of a conceptual relativization. Theory Soc..

[bib0048] Marshall A.T., Hackman D.A., Kan E., Abad S., Baker F.C., Baskin-Sommers A., Sowell E.R. (2022). Location matters: regional variation in association of community burden of COVID-19 with caregiver and youth worry. Health Place.

[bib0049] McQuaid R.J., Cox S.M., Ogunlana A., Jaworska N. (2021). The burden of loneliness: implications of the social determinants of health during COVID-19. Psychiatry Res..

[bib0050] Nkimbeng M., Nmezi N.A., Baker Z.G., Taylor J.L., Commodore-Mensah Y., Shippee T.P., Gaugler J.E. (2023). Depressive symptoms in older African migrants with mobility limitations: a descriptive study. Clin. Gerontol..

[bib0051] Palit S., Yang H., Li J., Khan M.A.S., Hasan M.J. (2022). The impact of the COVID-19 pandemic on the mental health of Rohingya refugees with pre-existing health problems in Bangladesh. Confl. Health.

[bib0052] Qu X., Qi X., Wu B., Yu J., Zhang H. (2023). Perceived social cohesion and depressive symptoms among internal migrants in China: the mediating role of social adaptation. Front. Public Health.

[bib71] Ringle C., Da Silva D., Bido D. (2015). Structural equation modeling with the SmartPLS. Rev. Bras. Mark..

[bib0054] Rapp C., Freitag M. (2015). Teaching tolerance? Associational diversity and tolerance formation. Polit. Stud..

[bib0055] Schiefer D., van der Noll J (2017). The essentials of social cohesion: a literature review. Soc. Indic. Res..

[bib0056] Scholten S., Velten J., Margraf J. (2018). Mental distress and perceived wealth, justice and freedom across eight countries: the invisible power of the macrosystem. PLoS One.

[bib0057] Scholten S., Velten J., Neher T., Margraf J. (2017). Wealth, justice and freedom: objective and subjective measures predicting poor mental health in a study across eight countries. SSM *Popul. Health*.

[bib0058] Schönfeld P., Brailovskaia J., Bieda A., Zhang X.C., Margraf J. (2016). The effects of daily stress on positive and negative mental health: mediation through self-efficacy. Int. J. Clin. Health Psychol..

[bib0059] Sert A., Elçi M., Uslu T., Şener İ. (2014). The effects of organizational justice and ethical climate on perceived work related stress. Procedia-Soc. Behav. Sci..

[bib0060] Silveira S., Hecht M., Matthaeus H., Adli M., Voelkle M.C., Singer T. (2022). Coping with the COVID-19 pandemic: perceived changes in psychological vulnerability, resilience and social cohesion before, during and after lockdown. Int. J. Environ. Res. Public Health.

[bib0062] Spiritus-Beerden E., Verelst A., Devlieger I., Langer Primdahl N., Botelho Guedes F., Chiarenza A., Derluyn I. (2021). Mental health of refugees and migrants during the COVID-19 pandemic: the role of experienced discrimination and daily stressors. Int. J. Environ. Res. Public Health.

[bib0063] Steel J.L., Dunlavy A.C., Harding C.E., Theorell T. (2017). The psychological consequences of pre-emigration trauma and post-migration stress in refugees and migrants from Africa. J. Immigr. Minor Health.

[bib0066] Staver A. (2013).

[bib0065] Sun J., Buys N., Stewart D., Shum D. (2020). Mediating effects of coping, personal belief, and social support on the relationship among stress, depression, and smoking behavior in university students. Health Educ..

[bib0067] Vaganian L., Boecker M., Bussmann S., Kusch M., Labouvie H., Margraf J., Gerlach A.L., Cwik J.C. (2022). Psychometric evaluation of the Positive Mental Health (PMH) scale using item response theory. BMC Psychiatry.

[bib0068] Velten J., Brailovskaia J., Margraf J. (2022). Positive Mental Health Scale: validation and measurement invariance across eight countries, genders, and age groups. Psychol. Assess..

[bib0069] Vukčević Marković M., Bobić A., Živanović M. (2023). The effects of traumatic experiences during transit and pushback on the mental health of refugees, asylum seekers, and migrants. Eur. J. Psychotraumatol..

[bib72] Werner E.A., Aloisio C.E., Butler A.D., D’Antonio K.M., Kenny J.M., Mitchell A., Monk C. (2020). Addressing mental health in patients and providers during the COVID-19 pandemic. Seminars Perinatol..

[bib70] Xiong R., Deng A. (2020). Prevalence and associated factors of postpartum depression among immigrant women in Guangzhou, China. BMC Pregnancy Childbirth.

